# Evidence of genetic structure in the wide-ranging bearded vulture (*Gypaetus barbatus* (Linnaeus, 1758))

**DOI:** 10.1186/s12862-021-01760-6

**Published:** 2021-03-15

**Authors:** Melanie Streicher, Sonja Krüger, Franziska Loercher, Sandi Willows-Munro

**Affiliations:** 1 Centre for Functional Biodiversity, School of Life Sciences, University of KwaZulu-Natal, Pietermaritzburg, South Africa; 2 Ezemvelo KZN Wildlife, Cascades, South Africa; 3 Stiftung Pro Bartgeier, Wuhrstrasse 12, CH-8003 Zuerich, Switzerland

**Keywords:** Bearded vulture, Gene flow, Habitat fragmentation, *Gypaetus barbatus*, Population decline, Genetic diversity

## Abstract

**Background:**

The bearded vulture is sparsely distributed across a wide geographic range that extends over three continents (Africa, Europe and Asia). Restriction to high-altitude mountainous habitats, low breeding rates, lack of food and a heightened level of persecution have left many local populations severely diminished or extinct. Understanding the genetic connectivity and population structure of this threatened vulture species is critical for accurately assessing their conservation status, and for appropriately managing local populations through captive breeding programmes or translocations. Previous genetic assessments of the species were mainly focused on the European and Asian populations and included limited representation of the geographically isolated southern African population. A single mitochondrial study, which focused on the African populations of the bearded vulture, detected limited genetic differentiation between populations in Ethiopia and southern Africa, with reduced haplotype diversity in the southern Africa population. In this study, we extend the previous genetic assessments of the species by examining the phylogeography and genetic connectivity of global *G. barbatus* populations using a panel of 14 microsatellite loci.

**Results:**

Analyses revealed spatially correlated genetic differentiation between regional populations and low levels of gene flow between these population fragments. In contrast to the mitochondrial data, the microsatellite data support the management of genetically different populations as separate entities.

**Conclusions:**

Low genetic diversity and geographic isolation are known to adversely affect the evolutionary potential of a species in the long-term. The high inbreeding found in the southern African *G. barbatus* and, to a lesser extent, the northern African populations highlights the need for conservation programmes to effectively manage populations of this species and maintain extant genetic diversity.

**Supplementary Information:**

The online version contains supplementary material available at 10.1186/s12862-021-01760-6.

## Background

Historically, bearded vultures, *Gypaetus barbatus*, were considerably more abundant, albeit always sparsely distributed across a wide area ranging from the Palearctic, through Afrotropic and into Indomalay regions [1]. In the past century however, populations of this species have become locally extinct, or survive in highly habitat-specific isolated refuges across their former range [2–4]. A population in the Pyrenees, the islands of Corsica and Crete, and a reintroduced population in the Alps currently comprise the European bearded vulture population; while southern Africa, Ethiopia and Morocco are the remaining habitats for the African bearded vultures [1, 2].

In Europe, the population declined in the nineteenth century due to food shortage and direct persecution. This led to local extinction in the Alps by the early twentieth century [5]. The Pyrenees is home to the largest population of bearded vultures in Europe, but this population has also declined severely [4, 6]. The populations on Sardinia (Italy) and in the Balkans became extinct in the 1960s and 2000s due to unintentional poisoning [7] and decreased food availability [8]. Vulture populations still occur on Corsica (France) and Crete (Greece) but these populations are small (less than 10 breeding pairs) and continued existence is precarious [8]. In 1986, the first birds from captive breeding were released back into the wild in the Alps. Currently (2019), the estimated population size in the Alps is 280–340 birds and 52 breeding pairs. The main threats for European bearded vultures remain non-intentional poisoning and collision with energy infrastructure [4, 9].

**Fig. 1:**
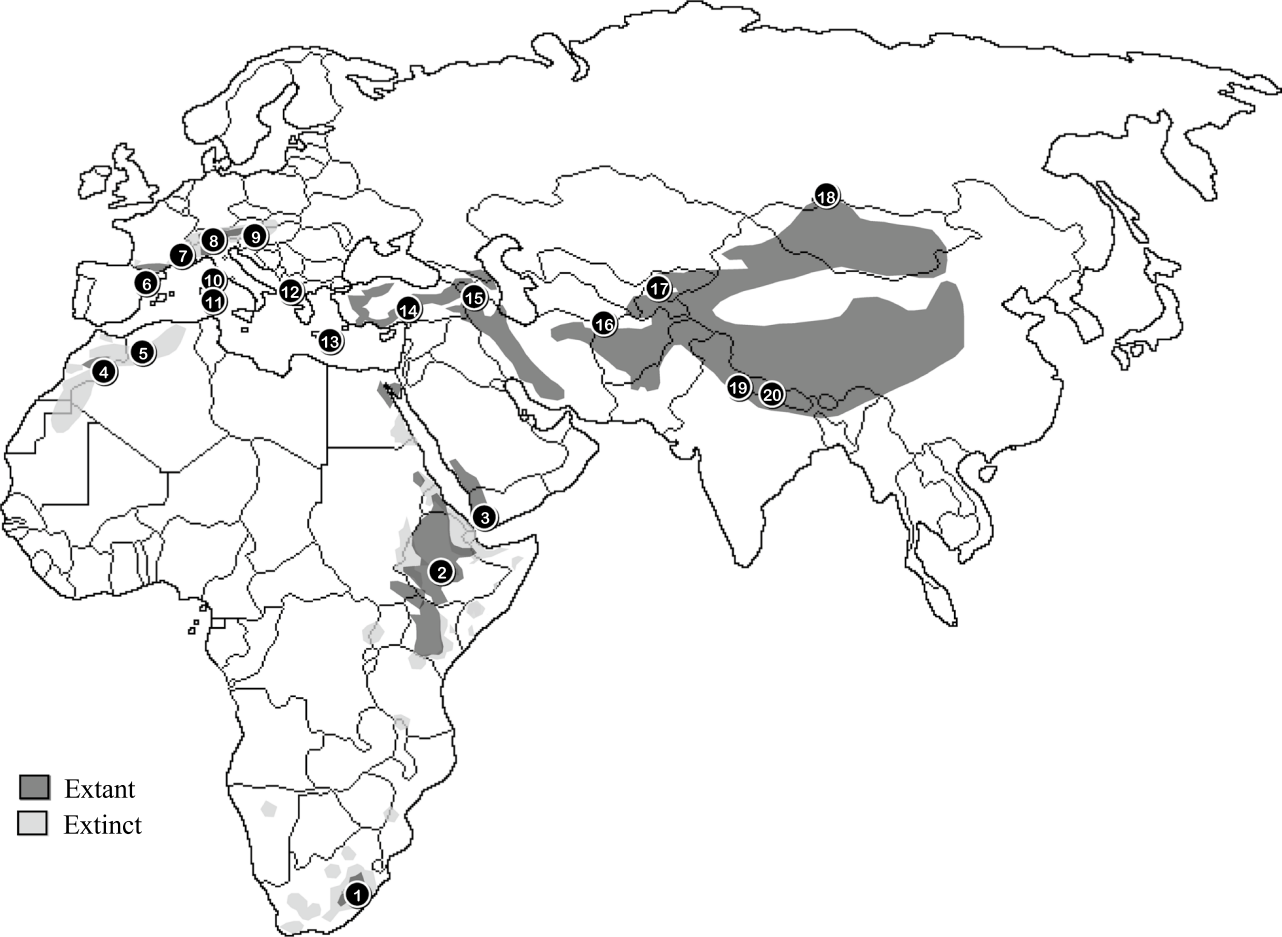
The bearded vulture’s *Gypaetus barbatus* global distribution (both extinct and extant) across the Palearctic, Afrotropic and Indomalay regions. Numbered circles correspond to broad sampling locality of individuals included in this study (Additional file 1: Table S4). Map created using ArcGIS and Adobe Illustrator

In Africa, habitat loss, electrocution and collision with energy infrastructure, unintentional poisoning through veterinary drug-use, feeding on poison baits aimed at predator control and intentional poisoning for the use of vulture body parts in the illegal wildlife and the traditional medicine trade, are the primary culprits for the population decline in many vulture species [10–16]. These threats have contributed to the fragmentation of the African bearded vulture population into several isolated pockets [10, 12, 15] (Fig. 1).

Morocco is home to fewer than 20 individuals and the species is considered extinct in Tunisia [17]. Ethiopia is believed to hold a few hundred pairs, however, reliable data are not available and these values are therefore thought to be considerably overestimated [18, 19]. A 2011-survey identified only three nest-sites in Kenya, and roughly double that in Tanzania [20]. The entire southern hemisphere population is restricted to the Highlands of Lesotho and south-eastern South Africa, where they roost and breed on high altitude cliffs of the Maloti-Drakensberg mountain border between South Africa (the Free State, KwaZulu-Natal and Eastern Cape Provinces) and Lesotho [2].

Due to the reduced effective population size, small disjunct populations experience amplified genetic drift, inbreeding and a greater loss of genetic diversity [21–23]. Loss of genetic variability directly impacts long-term persistence [24–27].

Two subspecies of bearded vulture have been recognized based on plumage characteristics and geography [28]—*Gypaetus barbatus barbatus* is distributed across Europe and Asia, extending into north-west Africa (mainly in High Atlas, Morocco), and *Gypaetus barbatus meridionalis* is found in north-east Africa, east Africa and extends into southern Africa. Studies based on maternally inherited mitochondrial data found evidence for two distinct lineages of bearded vultures which did not correspond geographically to the morphologically described subspecies. The first lineage described by Godoy et al. (2004) occurs predominantly in western Europe, and the other lineage extends over Africa, eastern Europe and central Asia. This study detected high levels of inter-population differentiation, with admixture between the two lineages occurring in Greece and the Alps [1]. Godoy et al. (2004) hypothesised the species to be genetically structured as a consequence of the extinction of the central European population. The two lineages underwent allopatric differentiation (Mediterranean vs. Africa/Asian), and range expansion in the African/Asian cohort which resulted in secondary contact zones in central Europe and northern Africa [1]. These contemporary, remnant populations were found to be ecologically interchangeable, even between vastly segregated populations. A further study including the southern African population of bearded vultures based on mitochondrial sequence data, found that, despite vast geographical distances separating populations, there is little genetic differentiation between the isolated southern African population and the northern hemisphere bearded vulture populations [29]. Most genetic studies have recorded low levels of genetic diversity in bearded vulture populations [1, 29, 30].

The genetic results have implications for captive breeding programmes and open the possibility for the translocation of individuals into shrinking populations. Unfortunately, previous studies have focussed their analyses almost entirely on the European population (with limited representation from southern Africa). The single study that included good representation of the isolated southern African population was based on mitochondrial DNA [29]. In this study we re-examine the population structure of the bearded vulture using improved sampling across the global distribution of the species and examining genetic connectivity among geographically isolated populations using a suite of biparentally inherited nuclear microsatellite markers. We test the hypothesis that the bearded vulture is a single, widely distributed species that shows shallow genetic structure despite the isolated nature of many populations. Estimating connectivity of disjunct populations will provide conservation authorities with the necessary information to form and implement the appropriate conservation measures to better protect local populations of the bearded vulture.

## Results

A total of 236 individuals were genotyped with the 14 microsatellite markers. All loci were polymorphic with the number of alleles per individual ranging from 6 (Gf11A4) to 17 (BV6, BV12). A summary of locus-by-locus measures of genetic diversity are provided in Additional file 1: Table S1. The mean null allele frequency was 18.9%. Paired t-tests, however, did not indicate a significant difference between uncorrected and corrected F_ST_ values (p-value > 0.05), suggesting that null alleles have a very limited effect on the genetic structuring analyses in these samples of bearded vultures. In addition, many of the analyses we conducted are not strongly influenced by the presence of null alleles [31]. Linkage disequilibrium analysis detected significant (p < 0.001) associations between some pairs of loci (Additional file 1: Table S4). Processes such as genetic drift, gene flow among populations with dissimilar allele frequencies and small sample size, can lead to erroneous detection of genetic association where there is none. Given that these microsatellites have been used in previous studies, all 14 microsatellite loci were used in subsequent analyses.

Significant departures (p < 0.05) from HWE were obtained for all loci in the southern African population. Loci 1, 3 and 4 were in HWE for the European, northern African and Eastern population complexes, respectively. The remaining loci did not conform to HWE predictions. This was not unexpected as these small isolated populations naturally deviate from the assumptions of HWE.

### Genetic diversity

The average number of alleles and effective alleles ranged from 5.93–7.00 and 2.81–3.23 respectively (Table 1). All populations tended towards being inbred (F_IS_ > 1, Table 1), and showed higher levels of homozygosity than would be expected under HWE (H_O_ < H_E_; Table 1).

**Table 1:** Genetic diversity estimates for 236 bearded vultures *Gypaetus barbatus*

Population	N	N_A_	N_E_	A_R_	A_P_	H_O_	uH_E_	F	F_IS_
Southern Africa	52	6.50	2.81	0.61	36	0.48	0.61	0.22	0.24
Northern Africa	39	6.36	3.08	0.57	8	0.40	0.56	0.30	0.30
Europe	107	7.00	3.23	0.55	18	0.48	0.55	0.13	0.23
Asia	38	5.93	2.97	0.54	3	0.42	0.54	0.23	0.23

The number of individuals (N), total number of alleles (N_A_), number of effective alleles (N_E_), allelic richness (A_R_), number of private alleles (A_P_), observed heterozygosity (H_O_), unbiased expected heterozygosity (uHe), fixation index (F), and inbreeding co-efficient (F_IS_) are given

### Population structure

**Fig. 2:**
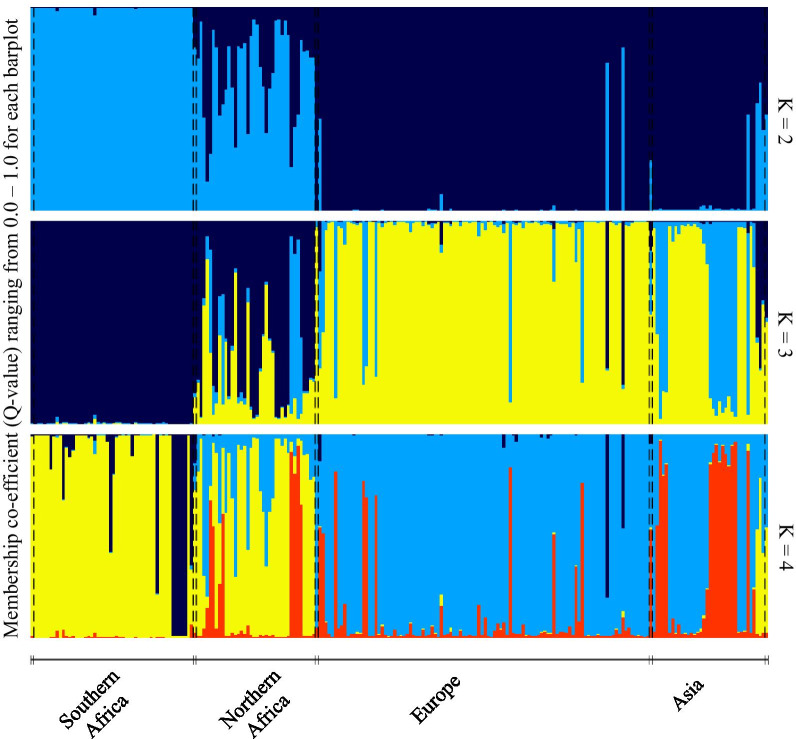
Probabilistic population structure under the *Admixed* model for 236 bearded vultures *Gypaetus barbatus* collected from across the global distribution of the species. Individuals are sorted into four broad geographically delineated regions, and are represented by vertical lines partitioned into coloured segments which represent the estimated membership coefficients (Q-value) of each cluster represented in their genetics. ∆K = 3 was estimated as the optimal partitioning strategy

The analysis using the *Admixture* parameter detected three genetic clusters (∆K = 3, Fig. 2) as the optimal partitioning scenario using both the Puechmaille and Evanno methods. The Bayesian STRUCTURE cluster plot revealed low levels of genetic admixture among populations from different geographic regions, and genetic clustering was closely linked to geography. The pattern of clustering does not support the delimitation of the two subspecies. The birds from Ethiopia and Yemen had alleles at similar frequencies to those seen in birds from north-west Africa, whereas the southern African population was distinct from other populations. For this reason, all northern African populations were grouped together in subsequent analyses.

The lowest admixture was evident in the European populations (Q = 0.97), followed by the southern African population (Q = 0.92) and the Asian populations (Q = 0.88). The highest admixture was detected in the northern African populations (Q = 0.63, Table 2). Results using the *No Admixture* parameter, returned a similar clustering pattern although in this case ∆K = 4. Barplots of K = 2 to 4 are shown in Fig. 2.

**Table 2:** Comparison of membership coefficients (Q-values) for both *Admixture* and *No Admixture* models used in STRUCTURE

Sample origin	Sample size	*Admixture*	*No Admixture*
Southern Africa	52	0.92	0.79
Northern Africa	39	0.63	0.72
Europe	107	0.97	0.94
Asia	38	0.88	0.79

In all analyses, the isolated southern African bearded vulture population is genetically distinct from all other bearded vulture populations. Genotypes collected from this region appear to belong to unique genetic clusters and are characterized by unique allele frequencies. The genetic distinctiveness of the southern African population is also reflected in the number of unique alleles found in this population (36 unique alleles). The European population complex contains only 18 unique alleles and the Asian and northern African complexes have fewer than 10 unique alleles each. Only 31 alleles are shared by all populations. Results from the Mantel test indicate that there was no positive correlation between genetic and geographical distance (r^2^ = 0.049; P < 0.001).

**Fig. 3:**
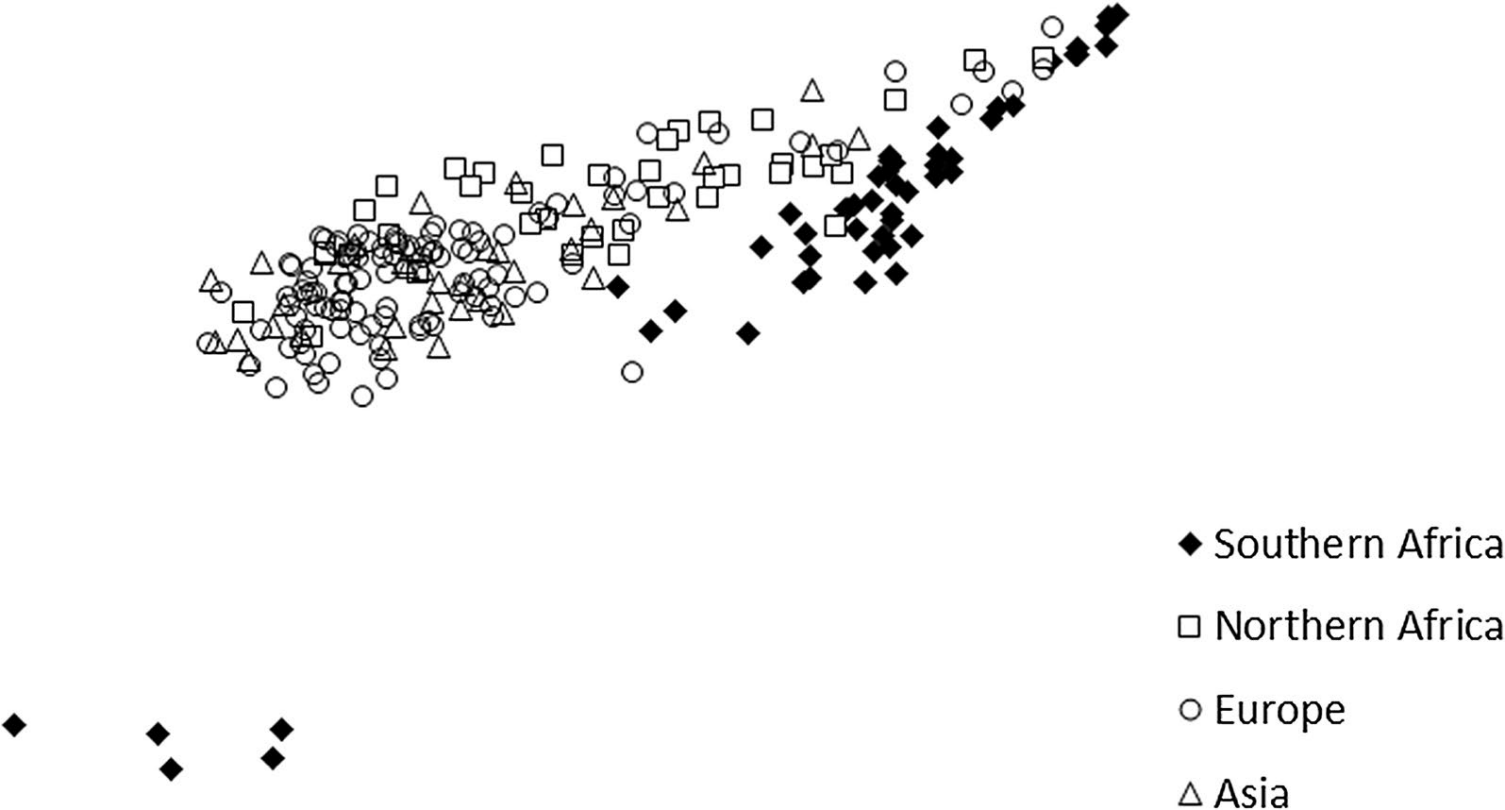
Population assignment of 236 bearded vultures *Gypaetus barbatus* across 14 microsatellite loci

Population assignment tests conducted in GenAlEx support the STRUCTURE results by showing the separation of the southern African population from the other African, European and Asian populations (Fig. 3). The latter three population complexes showed significant overlap and could not be separated in this analysis.

Pairwise F_ST_ comparisons only provide moderate support for differentiation of the southern African population from the northern African populations (F_ST_ = 0.12, Table 3) but indicate that the southern African population is genetically distinct from the European and Asian populations (F_ST_ = 0.18 and 0.16, respectively, Table 3).

**Table 3:** Pairwise F_ST_ among the four regional populations of bearded vulture *Gypaetus barbatus*

	Southern Africa	Northern Africa	Europe	Asia
Southern Africa	–	0.10	0.18	0.16
Northern Africa	0.12	–	0.11	0.09
Europe	0.20	0.12	–	0.03
Asia	0.18	0.10	0.03	–

Below the diagonal (indicated by hyphens) are uncorrected values, values above the diagonal are ENA-corrected values. Values < 0.05 indicate little genetic differentiation, 0.05–0.15 = moderate differentiation, 0.15–0.25 = great differentiation, and > 0.25 = very great differentiation

AMOVA results, conducted when grouping individuals into the four regions, showed that 49% (p < 0.001) of variation was attributed to within individual differences, while differences among individuals and among populations explained 39% (p < 0.001) and 12% (p < 0.001) of the diversity respectively.

### Population connectivity

The BayesAss 3.0 analysis indicates that the highest migration seems to have occurred from the European populations to the Asian populations (28.26%; 83 shared alleles). This supports the hypothesis that, before the extinction of bearded vultures in the Alps, there was an interconnected European population from Spain to the Alps, Balkans to Turkey and Asia.

Minimal migration has occurred within Africa in the recent past. Less migration took place from northern Africa to southern Africa, than the reverse (7.05% and 15.20%, Table 4). The northern African populations contain a greater percentage (15.64%, Table 4) of the European alleles than the southern African population (< 1%, Table 4), suggesting stronger linkage among northern hemisphere populations. However, alleles dominant in the Asian populations do occur, albeit at lower frequencies in both the southern and northern African populations (< 2%, Table 4). While the genetic admixture seen in the northern African bearded vulture populations may be a reflection of active migration, it is unlikely that the southern African population has experienced any recent genetic admixing with the Asian populations. Thus, this pattern may merely reflect incomplete lineage sorting.

**Table 4:** Pair-wise migration rates between the four regional population complexes; bold values on the diagonal show the proportion of population which are permanent residents

	Migrant % comprising resident population			
	Southern Africa	Northern Africa	Europe	Asia
Southern Africa	*90.17*	7.05	0.81	1.97
Northern Africa	15.20	*67.78*	15.64	1.38
Europe	0.40	0.92	*97.98*	0.70
Asia	1.79	1.09	28.26	*68.86*

**Fig. 4:**
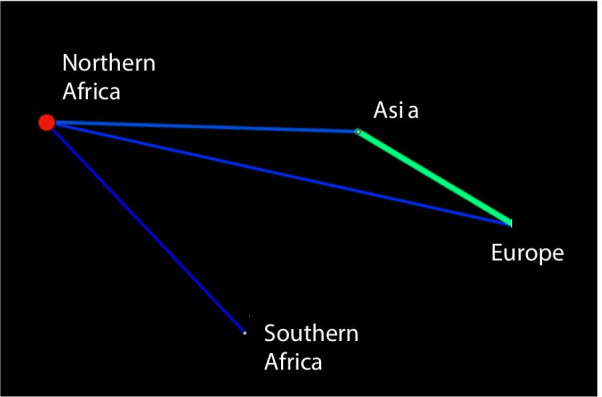
Network constructed in EDENetworks grouping 236 bearded vulture *Gypaetus barbatus* samples by region. Populations (nodes) are linked by edges which are weighted in proportion to the strength of the genetic linkage

The clustering patterns which emerge from the EDENetworks also indicates that the four regional population complexes are separate and that there has been limited recent connectivity. EDENetwork analysis reveals stronger linkage and overlap between Asian and European populations. The southern African population shares a weak linkage with the northern African populations but is not connected genetically with any of the other regional populations (Fig. 4). The northern African populations were connected (albeit weakly) with the Asian and European populations.

## Discussion

The population structure of endangered species reflects population collapses, bottlenecking events, fragmentation and isolation of populations [24, 32, 33]. A key indicator of population health is genetic variability, as healthy levels of variation allow for populations to respond to natural selection [27, 34, 35]. The associated loss of genetic variability in small isolated populations, through genetic drift and inbreeding, is believed to adversely affect the adaptive potential of remnant populations and thus threatens their evolutionary persistence [24, 36, 37]. Past genetic studies conducted on the wide-ranging but sparsely distributed bearded vulture have either focussed primarily on the European and Asian populations, and/or have used mitochondrial markers [1, 29]. These papers report a lack of mitochondrial genetic diversity within the species. Based on the lack of adequate mitochondrial differentiation between the geographically disjunct populations in these studies, and apparent ecological and genetic interchangeability, it was recommended that the southern and northern hemisphere populations of bearded vultures be managed as a single unit [1, 29]. Genetic structuring is often prevalent in populations which are widely distributed and display strong natal philopatric behaviour [30, 38, 39]. Given that the bearded vulture populations are separated by vast geographic distances, and philopatric behaviour has been documented in the species, it would be reasonable to expect genetic structuring [30, 40]. In the research presented here, population structuring was investigated by using biparentally inherited microsatellite fragment data.

The pattern of population differentiation found here provides clarification of the population structure and migration of bearded vultures. Although there is debate over the number of subspecies of bearded vultures [41–43], our results advocate for the management of the South African bearded vulture population as a separate entity, *G. b. meridionalis* (Keyserling & Blasius, 1840). There is debate over the splitting of the northern lineage into either one, *Gypaetus barbatus barbatus* [43, 44], or 3 subspecies, *Gypaetus barbatus aureus* (Hablizl, 1783), *Gypaetus barbatus hemachalanus* (Hutton, 1838) and *Gypaetus barbatus barbatus* (Linnaeus, 1758) [41, 42]. Based on our findings, we advocate recognition of *G. b. barbatus* in Eurasia and northern Africa and *G. b. meridionalis* in eastern and southern Africa. The diversification of the northern and southern bearded vulture lineages is hypothesised to be linked to the expanding Sahara Desert which acted as a physical barrier during the last glacial maximum [1, 45]. When the Sahara receded, bearded vultures were able to disperse into East Africa and this formed a ‘steppingstone’ allowing for admixture between the two subspecies. The highest level of admixture in this study was found in the northern African populations, and this could reflect past dispersal events, as well as the subsequent isolation in the recent past. Although there appears to be divergence between the SW Arabian and southern African population, further analysis based on a larger sample size from Yemen would be required to confirm this.

### Migration

Based on this species’ philopatric dispersal behaviour, genetic differentiation between regional populations is expected to increase with geographic distance following an isolation-by-distance model [46, 47], especially in the absence of translocations or supplementations to the population. Here analyses found minimal migration between the populations. The highest emigration rates were out of Europe and little to no migration was detected to, or from, the southern African population. This supports the hypothesis that the southern African population is indeed isolated, both geographically and genetically.

### Southern African bearded vulture diversity

Apart from past persecution, the southern African population of bearded vultures continues to suffer threats from several sources, adding to their continued decline [20, 48]. Past studies on the global and local bearded vulture populations have raised concern for their management based on the reduced genetic variability [1, 30, 48, 49]. Given that the southern African bearded vulture population is geographically and genetically isolated, it was expected that this population should be at risk genetically. Indeed, we found evidence for this and found the southern African bearded vulture population to harbour less genetic diversity than would be expected under Hardy–Weinberg, although this genetic diversity is still higher than that seen in other African vulture species [50]. The drastic population decline and reduced genetic variability of the bearded vulture places the southern African population alongside other ecologically and behaviourally similar vulture species impacted by “The African Vulture Crisis”. This population collapse, coupled with the isolated nature of the southern African population, raises concern for the long-term population health and persistence of the species in southern African. Genetic diversity levels will continue to decline if the population bottleneck is indefinitely sustained. The genetic variation seen in contemporary populations reflects the diversity in the previous generations. Should conservation seek to preserve this population’s gene-assemblage, active management would need to firstly minimise external threats, and secondly mitigate factors leading to population structuring, as this amplifies genetic drift.

This study highlights the utility of integrating genetic data to inform conservation strategies as it elucidates information that is not readily evident in the field. Our results suggest that the southern African bearded vulture population, being distinct from other such populations, may represent a reservoir of genetic variation that should be given separate conservation status.

## Methods

### Sampling

A total of 236 bearded vulture specimens were analysed in this study. These include samples from across the global distribution of the species (Fig. 1), and include historical as well as recent samples (1805–2012). Bearded vultures have a long generation time, and this temporal sampling represents approximately 10 generations. Unfortunately, the few historical samples preclude analysis of temporal changes in population structure. These samples were divided into four regional groupings for analyses: a southern African group, including 52 samples from a single population spanning South Africa and Lesotho; a northern African group (n = 39) including samples from Ethiopia, Yemen, Morocco and Algeria; a European group (n = 107) including samples from Albania, Greece, Turkey, Crete, Corsica, Sardinia, Switzerland, Austria, the Pyrenees, France, Spain; and an Asian group (n = 38) including samples from Caucasus, central Asia, Turkestan, Kyrgyzstan, Russia, India, and China. Details of specimens included in the study are provided in Additional file 1: Tables S1 and S5.

### DNA extraction

The NucleoSpin® Tissue kit (Macherey–Nagel) was used for all DNA extractions. The standard protocol for extracting genetic material from blood was followed for blood samples. The feather and archival tissue extractions were done using the standard protocol for muscle tissue, with the following modifications to increase DNA yield: incubation of the ~ 5 mm feather tip/skin snip and proteinase K for 48 h, lysate was then incubated in B3 (buffer) for 45 min (70 °C), the final volume of pre-warmed Buffer BE was decreased to 80 μl, incubation at 70 °C for 20 min followed by centrifuging and then reapplication of the solution onto the membrane, followed by incubation at 70 °C for an additional 5 min and a final centrifugation step. DNA concentrations were determined using the ThermoScientific NanoDrop 2000 spectrophotometer (Inqaba Biotec, South Africa). All DNA extracts were stored at -20 °C.

### Microsatellite amplification

Fourteen microsatellite loci (Table 5) were amplified in the current study. These microsatellite primers were developed specifically for European populations of bearded vulture [3] and for other *Gyps* species [51]. Microsatellite loci were amplified in four multiplex reactions, and one singleplex (annealing temperature for BV 17 excluded it from multiplexing) (Table 5) using KAPA2G Fast multiplex PCR Kit (KAPA Biosystems, Wilmington, MA, USA). PCR reactions consisted of 5 μl KAPA2G Fast Multiplex mix, 0.1- 0.2 μM of each primer, 0.5–3.5 μl of template DNA, and purified water was added to each reaction to make up a final reaction volume of 10 μl. The thermocycler cycling parameters were: 95 °C for 3 min as the initial denaturation step, 30 cycles at 95 °C for 15 s, 58–60 °C (Ta) for 30 s, 72 °C for 1 min, with a final elongation step at 72 °C for 10 min. The samples were held at 4 °C once the cycle had completed running. The amplified products were sent to the Central Analytical Facilities (Stellenbosch University, South Africa) for fragment analysis. To ensure correct genotype scoring, all genotypes from archival material were genotyped multiple times and 20% of newly collected samples were reamplified and compared to ensure consistency. Genotyping error was < 0.2%. Genotypes were scored using the software package GeneMarker® v2.4.0 (Soft Genetics).

**Table 5:** Microsatellite marker multiplex combinations, and associated information for primers used across populations of bearded vulture *Gypaetus barbatus*

Locus	Primer sequence (5′–3′)	Repeat motif	Ta (°C)	Label	Allele size range (bp)	Multiplex reaction
BV 9^a^	F: ATCTAGGGACATCGAGGAGC	(TA)6 (CA)11	58 °C	Hex	212	1
	R: ACAGGGATGCAGGTAAGCC					
BV14^a^	F: GGCAGTGTGGAGCCTACATC	(CA)16	58 °C	Fam	162–164	1
	R: CTCCAGGGTCCTTGTTTGC					
Gf11a4	F: GATCCCTTCCAACCGAAAAT	(CTCTT)17	58 °C	Hex	130–147	1
	R: TGGTGACCAACGGAAGTGTG					
BV2^a^	F: CAGCATGTTATTTTGGCTGC	(CA)11	60 °C	Hex	115–120	2
	R:TTGCTAAACCGGTTAGAAGTTG					
Gf8G	F: TGAGCAGGTGAGTCCAGAAG	(CT)8C(TC)2	60 °C	Fam	274	2
	R: GCTCTCCTGTCATCTTGCAT					
Gf3f3	F: GATCTTTCCCCTTCTGTG	(CT)10	60 °C	Tet	177–179	2
	R: TTCGTGCAGTGATGCTGGTG					
BV6^a^	F: AATCTGCATCCCAGTTCTGC	(CA)11	60 °C	Hex	106–119	3
	R: CCGGAGACTCTCAGAACTTAAC					
Gf3h3	F: GTAGAATAATTTGCTCCTGG	(CT)12	60 °C	Fam	137–141	3
	R: GTGAAGGCACCTCATAGACA					
Gf9C	F: GGTGGACATTACATACACTG	(TC)10 + (CT)9C (CA)5 T(AC)4	60 °C	Hex	262–268	3
	R: CAAGGAATCTGGACTACTAA					
BV5^a^	F: GTTCTGAGGGTAGAGGGACTG	(CA)17	58 °C	Tet	178	4
	R: GCTGAGCAGCTTCAGAAAGTC					
BV8^a^	F: TGGCATGCTGCTATGAGAAC	(CA)11	58°Cs	Fam	105	4
	R: GTGCTTTGCATGCTTTTACTC					
BV11^a^	F: TGTTTGCAAGCTGGAGACC	(CA)22	58 °C	Hex	160–162	4
	R: AAAAGCCTTGGGGTAAGCAC					
BV12^a^	F: TCAGGTTTTGACGACCTTCC	(CA)15	58 °C	Fam	256–269	4
	R: GTGGTAACGGAGGAACAAGC					
BV17^a^	F: TGATGTGCAGATGCGTGAC	(CA)11	62 °C	Hex	186	
	R: GGACTCTGATGAAGCCAAGC					

^a^Species-specific loci, the remaining are family specific

^b^BV17 was run in isolation

### Microsatellite analysis

Null alleles in data can be problematic for calculations of genetic variability as they can decrease within-population variance and so inflate F_ST_ values [52]. The presence of null alleles was tested for using FreeNA [52]. Uncorrected F_ST_ values were compared to F_ST_ values corrected using the excluding null alleles (ENA) method using a paired t-test. The presence of scoring errors, such as incorrect assigning of genotypes and fractions in genotypes, was assessed using GenAlEx v6.5 [53] and FSTAT [54]. These packages were also used to calculate the average number of alleles at each locus (N_A_), allelic richness (AR), inbreeding co-efficient (F_IS_) and gene diversity. Effective alleles at each locus (N_E_), observed (H_O_) and expected (H_E_) heterozygosity were calculated in GenAlEx. FSTAT was used to determine allelic richness and gene diversity. Tests for deviation from Hardy–Weinberg equilibrium (HWE) and linkage disequilibrium were performed in Genepop v4.2 [55] and FSTAT. The fixation indices, inbreeding co-efficient (F_IS_) and population differentiation (F_ST_) [56, 57] were calculated in FSTAT [58]. F_IS_ and F_ST_ explain the deviations seen from HWE. Positive F_IS_ values suggest higher inbreeding values (due to higher observed homozygosity) than would be expected under HWE, and vice versa. F_ST_ shows the genetic differentiation between two populations which no longer conform to the HWE assumptions.

### Population structure

Population structure across the global distribution of the bearded vulture was assessed using a Bayesian clustering method implemented in the software program STRUCTURE version 2.3.4 [59, 60]. The analysis was performed applying the correlated allele frequencies model [60] using sampling localities as prior information (LOCPRIOR parameter).

Analyses were run using both the *Admixture* and *No Admixture* model parameters. If there is reason to consider each population as completely discrete, the *No Admixture* model is appropriate. In contrast, the *Admixture* model allows for a large proportion of sampled individuals to have ancestry from multiple lineages, which implies a shallower population structure. The historically wide geographic distribution of the bearded vulture favours the *Admixture* model, and previous molecular work suggests that the African, European and Asian populations are genetically closely linked [1, 29]. The geographically isolated nature of these populations, however, suggests that each regional population may be acting as discrete units in recent generations. For this reason, both models were implemented and the output from both models presented.

All STRUCTURE analyses were performed with 100 000 Markov-Chain Monte Carlo (MCMC) replicates with a burnin of 10 000. The number of iterations for all assignments was 10 with K ranging from 1 to 10. Once all the simulations were completed, STRUCTURE Selector [61] was used to determine the optimum ∆K value for each assignment using the Puechmaille method [62]. Detecting the optimal number of genetic clusters was also conducted in STRUCTURE Harvester [63] using the Evanno method [64]. The online software package Pophelper [65] was used to compile bar plots of the optimum K- value (∆K).

Population differentiation (F_ST_) values were calculated in FreeNA using the ENA-corrected method. Population assignment analysis was conducted in GenAlEx. Pairwise F_ST_ values were calculated using two grouping schemes. Individuals were grouped by country of origin, and then because some countries have very low sample sizes, individuals were also grouped by region (southern Africa, northern Africa, Europe and Asia; Additional file 1: Table S1). Analysis of Molecular Variance (AMOVA) was performed after grouping individuals into the four geographic regions. Isolation-by-distance was tested for by assessing the correlation between genetic distance and geographic distance using a paired Mantel test [66] in GenAlEx.

### Regional connectivity

Regional connectivity was visualized through construction of a genetic network in EDENetworks v2.18 [67]. A genetic network with individuals grouped by region was constructed with edges or links between nodes weighted by pairwise F_ST_ genetic distances. The automatic thresholding option was used to determine the optimal percolation threshold. The final clustering pattern was recalculated 10 times to test for alternate placement of nodes or connections.

Recent migration among geographically segregated populations (southern Africa, northern Africa, Europe and Asia) was estimated using the Bayesian algorithm implemented in BayesAss 3.0 [68]. The analysis was conducted using the MCMC method with a burnin of 20 000 steps and a sampling frequency of 60 000 000 iterations as suggested by the authors of the programme [68]. The delta values for each parameter were adjusted to achieve a 20–60% acceptance rate [68]. A final delta value of 0.40 for allele frequency, migration and inbreeding rates maintained acceptance levels within limits. Migration rates below 0.10 were used to indicate demographic independent populations [69].

## Supplementary Information

**Additional file 1.** Supplementary data for Bearded Vulture analysis.

## Data Availability

The datasets generated and/or analysed during the current study are available in the Zenodo repository, https://doi.org/10.5281/zenodo.3859895.

## Abbreviations

Km: Kilometer
DNA: Deoxyribonucleic acid
F_ST_: Population differentiation
HWE: Hardy–Weinberg equilibrium
F_IS_: Inbreeding co-efficient
H_O_: Observed heterozygosity
H_E_: Expected heterozygosity
Q: Membership co-efficient
AMOVA: Analysis of molecular variance
ENA: Excluding null alleles
PCR: Polymerase chain reaction
MCMC: Markov-Chain Monte Carlo
