# Supplementary material for: Evidence of genetic structure in the wide-ranging bearded vulture (Gypaetus barbatus (Linnaeus, 1758))
Source: BMC Ecol Evol. 2021 Mar 15;21:42. doi: 10.1186/s12862-021-01760-6 (PMC7962245; doi:10.1186/s12862-021-01760-6)
Supplement: Supplementary file 1 — Additional file 1. Supplementary data for Bearded Vulture analysis. [file 12862_2021_1760_MOESM1_ESM.docx]

**Appendix**

**Table S1**

Segregation, origin and number of bearded vultures *Gypaetus barbatus* samples used for the STRUCTURE analysis; locality number given for indication on figures.

| Locality number | Regional grouping | Country/region of origin | Number of individuals |
| --- | --- | --- | --- |
| 1 | Southern Africa | South Africa, Lesotho | 52 |
| 2 | Northern Africa | Ethiopia | 24 |
| 3 | Northern Africa | Yemen | 7 |
| 4 | Northern Africa | Morocco | 1 |
| 5 | Northern Africa | Algeria | 7 |
| 6 | Europe | Spain | 6 |
| 7 | Europe | France | 11 |
| 8 | Europe | Switzerland | 35 |
| 9 | Europe | Austria | 1 |
| 10 | Europe | Corsica | 3 |
| 11 | Europe | Sardinia | 30 |
| 12 | Europe | Albania | 1 |
| 13 | Europe | Crete | 1 |
| 14 | Europe | Turkey | 1 |
| 15 | Asia | Caucasus | 7 |
| 16 | Asia | Turkestan | 5 |
| 17 | Asia | Kyrgyzstan | 2 |
| 18 | Asia | Russia | 15 |
| 19 | Asia | India | 8 |
| 20 | Asia | Tibet | 1 |

**Table S2**

Estimates of genetic diversity across 14 amplified loci in the southern African population of bearded vultures *Gypaetus barbatus;* where N_A_ is average number of alleles; N_E_ is number of effective alleles; H_O_ is observed heterozygosity; H_E_ is expected heterozygosity, A_R_ is Allelic richness and F_IS_ is gene diversity and inbreeding statistic.

| **Locus** | **Observed allele size** | | **N_A_** | | **N_E_** | | **H_O_** | | **H_E_** | | **A_R_** | | **Gene Diversity** | | **F_IS_** |
| --- | --- | --- | --- | --- | --- | --- | --- | --- | --- | --- | --- | --- | --- | --- | --- |
|  | | | |  | |  | |  | |  | |  | |  | |
| BV2 | | 108 - 136 | 7.25 | | 3.61 | | 0.54 | | 0.72 | | 5.94 | | 0.73 | | 0.26 |
| BV5 | | 154 - 194 | 9.00 | | 3.82 | | 0.41 | | 0.68 | | 7.25 | | 0.70 | | 0.49 |
| BV6 | | 105 - 201 | 8.75 | | 4.12 | | 0.50 | | 0.73 | | 7.11 | | 0.75 | | 0.31 |
| BV8 | | 103 - 117 | 3.25 | | 1.52 | | 0.17 | | 0.27 | | 2.73 | | 0.27 | | 0.32 |
| BV9 | | 135 - 221 | 5.25 | | 2.59 | | 0.72 | | 0.61 | | 4.25 | | 0.61 | | -0.17 |
| BV11 | | 143 - 193 | 8.75 | | 4.81 | | 0.53 | | 0.72 | | 7.46 | | 0.73 | | 0.33 |
| BV12 | | 231 - 271 | 9.75 | | 4.53 | | 0.85 | | 0.76 | | 7.75 | | 0.77 | | -0.11 |
| BV14 | | 157 - 191 | 8.50 | | 4.46 | | 0.88 | | 0.76 | | 6.61 | | 0.77 | | -0.15 |
| BV17 | | 183 - 197 | 3.75 | | 1.73 | | 0.34 | | 0.39 | | 3.26 | | 0.39 | | 0.17 |
| Gf3f3 | | 138 - 180 | 4.25 | | 2.00 | | 0.15 | | 0.49 | | 3.37 | | 0.51 | | 0.75 |
| Gf3h3 | | 111 - 189 | 6.50 | | 3.10 | | 0.55 | | 0.64 | | 5.29 | | 0.65 | | 0.16 |
| Gf8g | | 164 - 280 | 4.50 | | 1.37 | | 0.12 | | 0.20 | | 2.86 | | 0.20 | | 0.56 |
| Gf9c | | 206 -268 | 7.25 | | 3.33 | | 0.28 | | 0.67 | | 5.16 | | 0.68 | | 0.59 |
| Gf11a4 | | 125 - 160 | 3.50 | | 1.29 | | 0.16 | | 0.17 | | 2.42 | | 0.17 | | 0.17 |
| Total | |  | 6.45 | | 3.02 | | 0.44 | | 0.56 | | 5.11 | | 0.57 | | 0.26 |

#
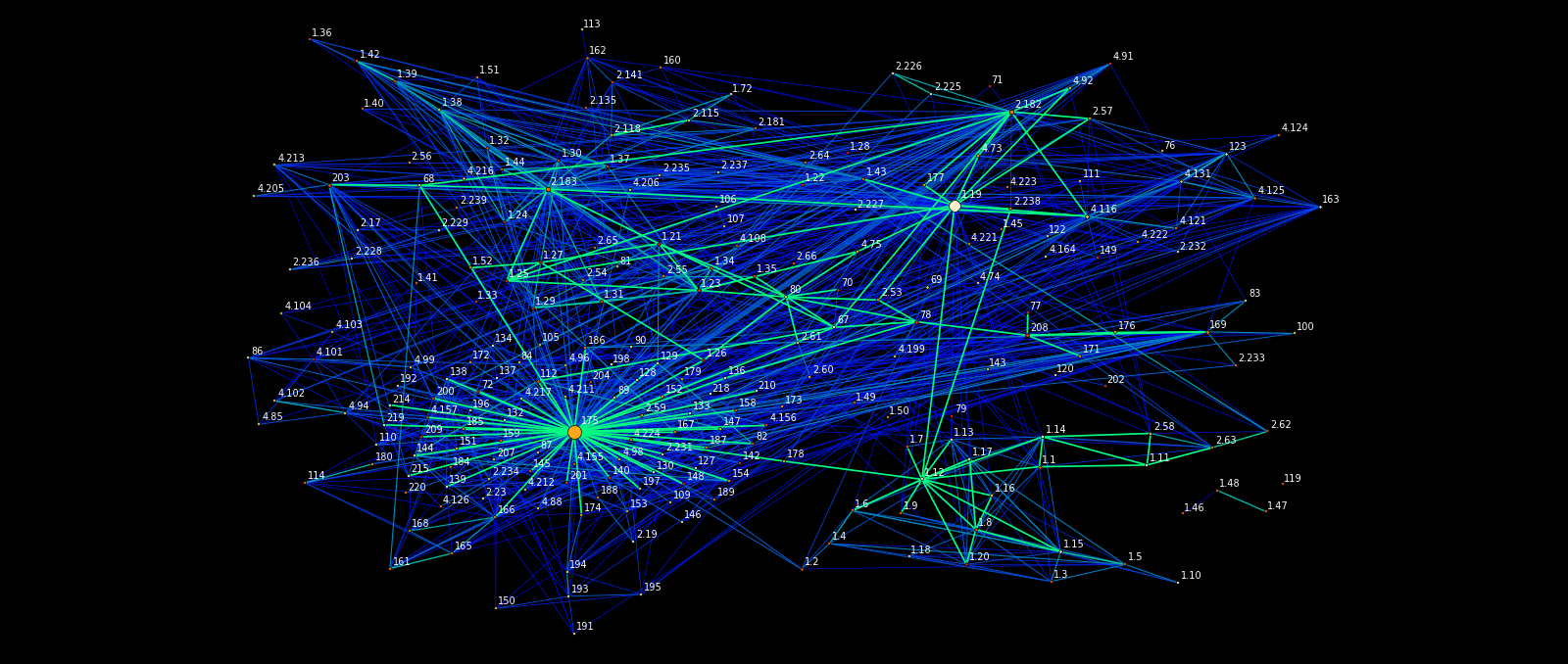


Appendix Fig. 1 Network constructed of all individual bearded vultures *Gypaetus barbatus* (n = 236) in EDENetworks. Populations (nodes) are named based on broad geographic region and are linked by edges which are weighted in proportion to the strength of the genetic linkage. Individuals are named according to their population, ie. 4.126 is individual 126 from population 4 (1- southern Africa, 2- northern Africa, 3- Europe, 4- Asia).


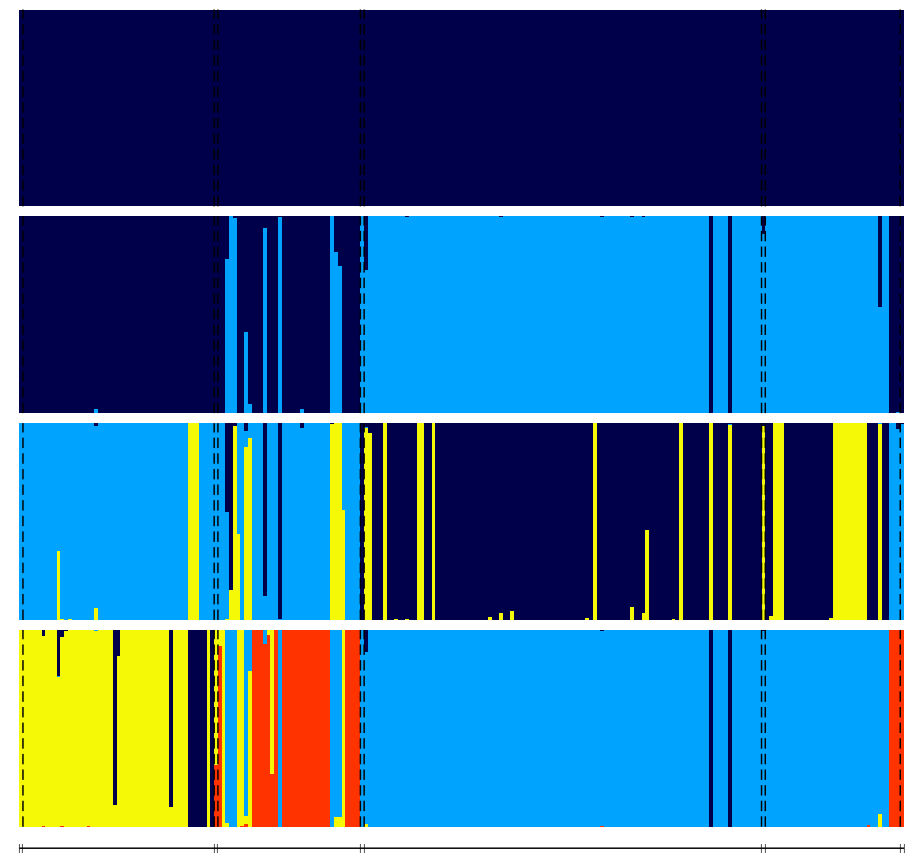

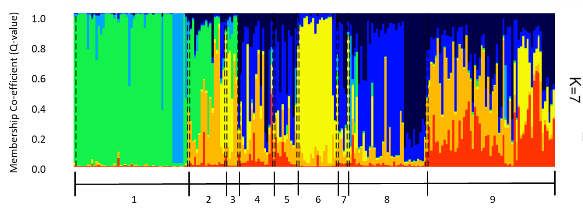

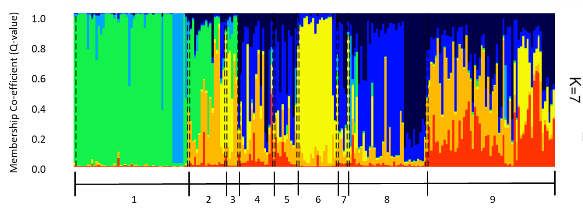

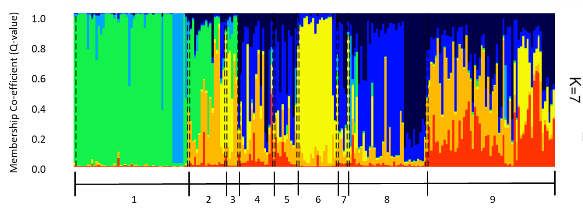


Southern Africa

Northern Africa

Europe

Asia

Appendix Fig. 2 Probabilistic population structure under the *No Admixture* model given for 236 bearded vultures *Gypaetus barbatus* collected from across the global distribution of the species. Individual vultures have been grouped into broad geographically delineated regions along the x-axis, and are represented by vertical lines which are partitioned into (K = 2 - 4) coloured segments which represent the estimated membership coefficients (Q-value) of each cluster represented in their genetics.

**Table S3**

Summary statistics of the 14 microsatellite loci amplified from 236 bearded vultures *Gypaetus barbatus*. Percentage missing data (MD), number of alleles (N_A_), null allele frequency (No), fixation index (F_ST_), observed heterozygosity (Ho), unbiased expected heterozygosity (uHe), deviation from Hardy-Weinberg (HWD).

| **Locus** | **MD** | **N_A_** | **N_O_** | **F_ST_** | **H_O_** | **uH_E_** | **HWD** |
| --- | --- | --- | --- | --- | --- | --- | --- |
| BV2 | 11 | 7.25 | 0.21 | 0.33 | 0.54 | 0.85 | *** |
| BV5 | 32 | 9.00 | 0.23 | 0.32 | 0.41 | 0.81 | *** |
| BV6 | 18 | 8.75 | 0.20 | 0.35 | 0.50 | 0.80 | *** |
| BV8 | 13 | 3.25 | 0.24 | 0.41 | 0.17 | 0.29 | *** |
| BV9 | 15 | 5.25 | 0.04 | -0.25 | 0.72 | 0.61 | *** |
| BV11 | 25 | 8.75 | 0.14 | 0.27 | 0.53 | 0.85 | *** |
| BV12 | 16 | 9.75 | 0.03 | 0.001 | 0.85 | 0.88 | *** |
| BV14 | 13 | 8.50 | 0.02 | -0.09 | 0.88 | 0.83 | *** |
| BV17 | 11 | 3.75 | 0.13 | 0.17 | 0.34 | 0.49 | *** |
| Gf3f3 | 14 | 4.25 | 0.44 | 0.77 | 0.15 | 0.57 | *** |
| Gf3h3 | 34 | 6.50 | 0.16 | 0.25 | 0.55 | 0.69 | *** |
| Gf8g | 16 | 4.50 | 0.33 | 0.50 | 0.12 | 0.18 | *** |
| Gf9c | 13 | 7.25 | 0.38 | 0.61 | 0.28 | 0.68 | *** |
| Gf11a4 | 16 | 3.50 | 0.12 | 0.25 | 0.16 | 0.15 | *** |
| Total |  | 6.45 | 0.19 | 0.28 | 0.44 | 0.62 | *** |

*** P<0.001

**Table S4**

Linkage disequilibrium per pair of loci with associated p-values

| BV9 x Gf11a4 | 0.003 | Gf11a4 x BV12 | 0.502 | Gf3f3 x Gf8g | **0.001** | BV6 x BV17 | 0.024 |
| --- | --- | --- | --- | --- | --- | --- | --- |
| BV9 x BV14 | **0.001** | Gf11a4 x BV17 | 0.154 | Gf3f3 x BV6 | **0.001** | Gf3h3 x Gf9c | 0.015 |
| BV9 x BV2 | **0.001** | BV14 x BV2 | **0.001** | Gf3f3 x Gf3h3 | 0.002 | Gf3h3 x BV5 | 0.224 |
| BV9 x Gf3f3 | 0.005 | BV14 x Gf3f3 | **0.001** | Gf3f3 x Gf9c | **0.001** | Gf3h3 x BV8 | **0.001** |
| BV9 x Gf8g | **0.001** | BV14 x Gf8g | 0.005 | Gf3f3 x BV5 | 0.002 | Gf3h3 x BV11 | **0.001** |
| BV9 x BV6 | 0.049 | BV14 x BV6 | **0.001** | Gf3f3 x BV8 | **0.001** | Gf3h3 x BV12 | 0.057 |
| BV9 x Gf3h3 | **0.001** | BV14 x Gf3h3 | 0.012 | Gf3f3 x BV11 | 0.009 | Gf3h3 x BV17 | **0.001** |
| BV9 x Gf9c | **0.001** | BV14 x Gf9c | 0.008 | Gf3f3 x BV12 | 0.099 | Gf9c x BV5 | **0.001** |
| BV9 x BV5 | 0.013 | BV14 x BV5 | 0.018 | Gf3f3 x BV17 | **0.001** | Gf9c x BV8 | **0.001** |
| BV9 x BV8 | **0.001** | BV14 x BV8 | 0.002 | Gf8g x BV6 | 0.171 | Gf9c x BV11 | **0.001** |
| BV9 x BV11 | 0.008 | BV14 x BV11 | 0.002 | Gf8g x Gf3h3 | **0.001** | Gf9c x BV12 | 0.097 |
| BV9 x BV12 | 0.012 | BV14 x BV12 | **0.001** | Gf8g x Gf9c | **0.001** | Gf9c x BV17 | **0.001** |
| BV9 x BV17 | **0.001** | BV14 x BV17 | **0.001** | Gf8g x BV5 | 0.290 | BV5 x BV8 | **0.001** |
| Gf11a4 x BV14 | 0.198 | BV2 x Gf3f3 | **0.001** | Gf8g x BV8 | **0.001** | BV5 x BV11 | **0.001** |
| Gf11a4 x BV2 | 0.882 | BV2 x Gf8g | 0.004 | Gf8g x BV11 | 0.003 | BV5 x BV12 | 0.296 |
| Gf11a4 x Gf3f3 | **0.001** | BV2 x BV6 | 0.027 | Gf8g x BV12 | 0.059 | BV5 x BV17 | 0.006 |
| Gf11a4 x Gf8g | 0.235 | BV2 x Gf3h3 | 0.002 | Gf8g x BV17 | **0.001** | BV8 x BV11 | **0.001** |
| Gf11a4 x BV6 | 0.392 | BV2 x Gf9c | **0.001** | BV6 x Gf3h3 | 0.078 | BV8 x BV12 | 0.018 |
| Gf11a4 x Gf3h3 | 0.805 | BV2 x BV5 | 0.245 | BV6 x Gf9c | 0.024 | BV8 x BV17 | **0.001** |
| Gf11a4 x Gf9c | 0.016 | BV2 x BV8 | 0.026 | BV6 x BV5 | 0.689 | BV11 x BV12 | 0.210 |
| Gf11a4 x BV5 | 0.511 | BV2 x BV11 | 0.002 | BV6 x BV8 | 0.185 | BV11 x BV17 | **0.001** |
| Gf11a4 x BV8 | 0.020 | BV2 x BV12 | **0.001** | BV6 x BV11 | 0.326 | BV12 x BV17 | 0.009 |
| Gf11a4 x BV11 | 0.289 | BV2 x BV17 | **0.001** | BV6 x BV12 | 0.005 |  |  |

**Table S5**

Details of 236 bearded vulture *Gypaetus barbatus* samples used in the study. Samples were collected in the field by Sonja Krüger (SK) or were sampled from museum collection: Durban Natural Sciences Museum (DNSM, South Africa), Natural History Museum, Tring (NHMT, United Kingdom), Naturalis Biodiversity Centre (RMNH, Netherlands), Stiftung Pro Bartgeier (SPB) Switzerland, and American Museum of Natural History (AMNH, United States).

| Provided by | Sample name | | Storage Facility Catalogue no. | Broad locality | Precise locality if available | Collection date |  |
| --- | --- | --- | --- | --- | --- | --- | --- |
| AMNH | | A1 | 268806 | Ethiopia | Kaka Mt., Arussi | 1929 | |
| AMNH | | A4 | 535811 | Sardinia | Urgulei, Ogliastra | 1902 | |
| AMNH | | A5 | 535812 | Sardinia | Iglesias | 1905 | |
| AMNH | | A6 | 535813 | Sardinia | Ascuentu (Guspini) | 1903 | |
| AMNH | | A7 | 535815 | Sardinia | Aritzo | 1903 | |
| AMNH | | A8 | 535817 | Greece | Parnes, Attica | 1900 | |
| AMNH | | A9 | 535821 | Greece | Parnass, Velitza | 1900 | |
| AMNH | | A11 | 535824 | Russia | Goudan, Transcaspian | 1900 | |
| AMNH | | A12 | 535826 | Kyrgyzstan | Tian-Shan Mts. | 1911 | |
| AMNH | | A13 | 535830 | Yemen | Sôk al Khamis | 1913 | |
| AMNH | | A14 | 535829 | Yemen | Sôk al Khamis | 1913 | |
| AMNH | | A16 | 535832 | India | Simla, N.W. Himilayas | 1913 | |
| AMNH | | A17 | 535836 | Morocco | Djebel Tixa (Atlas) | 1905 | |
| AMNH | | A18 | 535837 | Algeria | Djebel Taya, Meskoutine | 1909 | |
| AMNH | | A19 | 535839 | Algeria | Kerrata | 1904 | |
| AMNH | | A20 | 535840 | Algeria | El Kantara | 1909 | |
| AMNH | | A21 | 535848 | Ethiopia | Yeka Hill, Addis Ababa | 1926 | |
| AMNH | | A22 | 535855 | Ethiopia | Addis Ababa | 1914 | |
| AMNH | | A23 | 535856 | Ethiopia | ddis Ababa | 1914 | |
| AMNH | | A25 | 535858 | Ethiopia | Addis Ababa | 1914 | |
| UKZN | | BV001 | G22911 | South Africa | Southern Drakensberg | 2006 | |
| UKZN | | BV002 | G27343 | South Africa | Southern Drakensberg | 2007 | |
| UKZN | | BV003 | G27341 | South Africa | Southern Drakensberg | 2007 | |
| UKZN | | BV004 | G27307 | South Africa | Southern Drakensberg | 2009 | |
| UKZN | | BV005 | G27313 | South Africa | Southern Drakensberg | 2010 | |
| UKZN | | BV006 | G27306 | South Africa | Southern Drakensberg | 2009 | |
| UKZN | | BV007 | G27378 | Lesotho | Lesotho | 2012 | |
| UKZN | | BV008 | G27308 | South Africa | Southern Drakensberg | 2009 | |
| UKZN | | BV009 | G27376 | South Africa | Northern Drakensberg | 2011 | |
| UKZN | | BV010 | G27314 | South Africa | Northern Drakensberg | 2010 | |
| UKZN | | BV011 | BV14 inside | South Africa | NE Free State | 2004 | |
| UKZN | | SBV012 | BV2002 | South Africa | Central Drakensberg | 2002 | |
| UKZN | | BV013 | G27302 | Lesotho | Lesotho | 2008 | |
| UKZN | | BV014 | BV2009 | South Africa | Central Drakensberg | 2009 | |
| UKZN | | BV015 | G27375 | South Africa | Southern Drakensberg | 2011 | |
| UKZN | | BV016 | G27305 | South Africa | Southern Drakensberg | 2009 | |
| UKZN | | BV017 | G27303 | Lesotho | Lesotho | 2008 | |
| UKZN | | BV018 | G27309 | South Africa | Southern Drakensberg | 2009 | |
| UKZN | | BV019 | 12 | South Africa | Southern Drakensberg | 2003 | |
| UKZN | | BV020 | 6 | Lesotho | Lesotho | Not provided | |
| UKZN | | BV021 | 25 | South Africa | Northern Drakensberg | 2011 | |
| UKZN | | BV022 | 13 | South Africa | Central Drakensberg | 2008 | |
| UKZN | | BV023 | 19 | South Africa | Northern Drakensberg | 2009 | |
| UKZN | | BV024 | 27 | South Africa | Southern Drakensberg | 2010 | |
| UKZN | | BV025 | 11 | South Africa | Southern Drakensberg | 2000 | |
| UKZN | | BV026 | 10 | South Africa | Southern Drakensberg | 2007 | |
| UKZN | | BV027 | 31.1 | South Africa | Southern Drakensberg | 2010 | |
| UKZN | | BV028 | 31.2 | South Africa | Southern Drakensberg | 2010 | |
| UKZN | | BV029 | 30 | South Africa | Northern Drakensberg | 2010 | |
| UKZN | | BV030 | 9 | South Africa | Southern Drakensberg | 2009 | |
| UKZN | | BV034 | G27382 | South Africa | Central Drakensberg | 2012 | |
| UKZN | | BV035 | 2.8.12 | South Africa | Eastern Cape | 2012 | |
| UKZN | | BV036 | G27377 | South Africa | Northern Drakensberg | 2012 | |
| UKZN | | BV037 | G27379 | South Africa | Central Drakensberg | 2012 | |
| UKZN | | BV038 | G27381 | South Africa | Northern Drakensberg | 2012 | |
| UKZN | | BV039 | G27383 | South Africa | Central Drakensberg | 2012 | |
| UKZN | | BV040 | G27384 | South Africa | Central Drakensberg | 2012 | |
| UKZN | | BV031 | Kolo | Ethiopia | Not provided | 2001 | |
| UKZN | | BV032 | Mutt | Ethiopia | Not provided | 2001 | |
| UKZN | | BV033 | Putin | Ethiopia | Not provided | 2001 | |
| UKZN | | G27380 | G27380 | South Africa | Not provided | 2011 | |
| UKZN | | 34 | 34 | South Africa | Not provided | Not provided | |
| UKZN | | G27319 | G27319 | South Africa | Northern Drakensberg | 2010 | |
| UKZN | | G27310 | G27310 | South Africa | Central Drakensberg | 2010 | |
| UKZN | | G27311 | G27311 | South Africa | Northern Drakensberg | 2010 | |
| UKZN | | G27315 | G27315 | South Africa | Northern Drakensberg | 2010 | |
| UKZN | | G27316 | G27316 | South Africa | Northern Drakensberg | 2010 | |
| UKZN | | G27342 | G27342 | South Africa | Southern Drakensberg | 2007 | |
| UKZN | | BV6 | BV6 | Lesotho | Lesotho | 2009 | |
| UKZN | | BV7 | BV7 | Lesotho | Lesotho | 2009 | |
| UKZN | | BV8 | BV8 | Lesotho | Lesotho | 2009 | |
| UKZN | | BV10 | BV10 | Lesotho | Lesotho | 2009 | |
| UKZN | | BV12 | BV12 | Lesotho | Lesotho | 2009 | |
| UKZN | | g | g | South Africa | Drakensberg | Not provided | |
| DNSM | |  | DNSM 2 | South Africa | KZN, Kamberg | 1961 | |
| RMNH | | L1 | L1 | Switzerland | Switzerland | Not provided | |
| RMNH | | L2 | L2 | Sardinia | Sardinia | Not provided | |
| RMNH | | L3 | L3 | Sardinia | Sardinia | Not provided | |
| RMNH | | L4 | L4 | Sardinia | Sardinia | Not provided | |
| RMNH | | L5 | L5 | Pyrenees | Not provided | Not provided | |
| RMNH | | L6 | L6 | Turkey | Not provided | Not provided | |
| RMNH | | L7 | L7 | Greece | Not provided | Not provided | |
| SPB | | SB1 | BG178 | Greece | Greece | Not provided | |
| SPB | | SB2 | BG725 | Pyrenees | Pyrenees | Not provided | |
| SPB | | SB3 | BG131 | Russia | Former Soviet Union | Not provided | |
| SPB | | SB4 | BG680 | Pyrenees | Aragon Pyrenean | Not provided | |
| SPB | | SB5 | BG065 | Crete | Crete | Not provided | |
| SPB | | SB6 | BG132 | Russia | Former Soviet Union | Not provided | |
| SPB | | SB7 | BG652 | Pyrenees | Aragon Pyrenees | Not provided | |
| SPB | | SB8 | BG551 | Pyrenees | Spanish Pyrenees | Not provided | |
| SPB | | SB9 | BG151 | Russia | Former Soviet Union | Not provided | |
| SPB | | SB10 | BG482 | Kyrgyzstan | Kyrgyzstan | Not provided | |
| SPB | | SB13 | BG022 | Russia | Former Soviet Union | Not provided | |
| SPB | | SB16 | BG201 | Turkestan | Tadzhikistan | Not provided | |
| SPB | | SB18 | BG003 | Central Asia | West-middle Asia | Not provided | |
| SPB | | SB19 | BG199 | Russia | Former Soviet Union | Not provided | |
| SPB | | SB20 | BG232 | Pyrenees | Spanish Pyrenees | Not provided | |
| SPB | | SB21 | BG014 | Russia | Not Provided | Not provided | |
| SPB | | SB22 | BG204 | Russia | Not provided | Not provided | |
| SPB | | SB23 | BG153 | Russia | Not provided | Not provided | |
| SPB | | SB24 | BG009 | Russia | Not provided | Not provided | |
| SPB | | S1.1 | 1 | Spain | Sierra Magina | Not provided | |
| SPB | | S1.2 | 2 | Spain | Sierra Magina | 1930 | |
| SPB | | S1.3 | 3 | Switzerland | Glarus | 1830 | |
| SPB | | S1.4 | 4 | Caucasus | Caucasus, North Side | 1900 | |
| SPB | | S1.6 | 6 | Greece | Greece | 1905 | |
| SPB | | S1.7 | 7 | Pyrenees | Pyrenees | 1904 | |
| SPB | | S1.9 | 9 | Switzerland | Basel Zoo | 1987 | |
| SPB | | S1.10 | 10 | Switzerland | Oberhasli | 1805 | |
| SPB | | S1.11 | 11 | Switzerland | Brienzersee | 1823 | |
| SPB | | S1.12 | 12 | Switzerland | Zuoz | 1854 | |
| SPB | | S1.14 | 14 | Algeria | Algeria | Not provided | |
| SPB | | S1.15 | 15 | Russia | Russia | 1979 | |
| SPB | | S1.16 | 16 | Ethiopia | Abyssinia | 1959 | |
| SPB | | S1.17 | 17 | Ethiopia | Abyssinia | 1959 | |
| SPB | | S1.18 | 18 | Corsica | Asco, Haute Corse | 1957 | |
| SPB | | S1.19 | 19 | Albania | Lamentite | 1920 | |
| SPB | | S1.20 | 20 | Caucasus | Petrowsk | 1900 | |
| SPB | | S1.24 | 24 | Greece | Parnio Chasia Ciocha | 1899 | |
| SPB | | S1.25 | 25 | Greece | Parnis Chasia Ciocha | 1899 | |
| SPB | | S1.26 | 26 | India | Khalatase Ladakh | 1929 | |
| SPB | | S1.27 | 27 | India | Ladka | 1930 | |
| SPB | | S1.29 | 29 | Caucasus | Nislam | 1900 | |
| SPB | | S1.30 | 30 | Sardinia | Ogliastra | 1902 | |
| SPB | | S1.31 | 31 | Sardinia | Sardinia | 1905 | |
| SPB | | S1.32 | 32 | Sardinia | Sardinia | 1901 | |
| SPB | | S1.33 | 33 | Sardinia | Uzzulei | 1911 | |
| SPB | | S1.36 | 36 | Central Asia | Thian Shan | 1900 | |
| SPB | | S1.39 | 39 | France | Bagnerre de bigore | Not provided | |
| SPB | | S1.43 | 43 | Spain | South Spain "gaitanes" | 1869 | |
| SPB | | S1.44 | 44 | Switzerland | Ftan, Val Tasua | 1859 | |
| SPB | | S1.50 | 50 | Ethiopia | Ethiopia | 1887 | |
| SPB | | S1.51 | 51 | Sardinia | Sardegna | Not provided | |
| SPB | | S1.52 | 52 | Sardinia | Colle di Tena, Conoraza | 1885 | |
| SPB | | S1.54 | 54 | Sardinia | Sardegna | 1901 | |
| SPB | | S1.56 | 56 | Sardinia | Sardegna | 1907 | |
| SPB | | S1.57 | 57 | Sardinia | Sardegna | 1907 | |
| SPB | | S1.60 | 60 | Ethiopia | Abyssinia | 1900 | |
| SPB | | S1.61 | 61 | Greece | Greece | 1899 | |
| SPB | | S1.63 | 63 | Spain | Sierra | 1899 | |
| SPB | | S1.66 | 66 | Pyrenees | Tardet | 1896 | |
| SPB | | S1.71 | 71 | Sardinia | Sardinia | 1906 | |
| SPB | | S1.73 | 73 | France | Presles. Pont en Royans | Not provided | |
| SPB | | S1.74 | 74 | Spain | Sierra Magina | 1920 | |
| SPB | | S1.77 | 77 | Switzerland | Tessin | 1903 | |
| SPB | | S1.78 | 78 | Switzerland | Zermatt, Wallis | 1839 | |
| SPB | | S1.79 | 79 | Switzerland | Wallis | 1886 | |
| SPB | | S1.80 | 80 | Sardinia | Not provided | 1915 | |
| SPB | | S1.81 | 81 | Spain | Not provided | 1940 | |
| SPB | | S1.84 | 84 | Switzerland | Oberland | 1850 | |
| SPB | | S1.85 | 85 | Switzerland | Brig, Wallis | 1833 | |
| SPB | | S1.88 | 88 | Caucasus | Caucasus | 1905 | |
| SPB | | S1.89 | 89 | Turkestan | Naryn | 1913 | |
| SPB | | S1.90 | 90 | Turkestan | Naryn | 1910 | |
| SPB | | S2.92 | 92 | Switzerland | Switzerland | 1881 | |
| SPB | | S2.93 | 93 | Switzerland | Switzerland | 1891 | |
| SPB | | S2.95 | 95 | Switzerland | Not provided | 1981 | |
| SPB | | S2.98 | 98 | Greece | Naundes, Tirol | 1871 | |
| SPB | | S2.99 | 99 | Greece | Parnassus | 1885 | |
| SPB | | S2.101 | 101 | Sardinia | Not provided | 1917 | |
| SPB | | S2.105 | 105 | Caucasus | Caucasus | 1904 | |
| SPB | | S2.106 | 106 | Austria | Vorarlberg | Not provided | |
| SPB | | S2.107 | 107 | Sardinia | Not provided | Not provided | |
| SPB | | S2.109 | 109 | Switzerland | Andeer | Not provided | |
| SPB | | S2.110 | 110 | Switzerland | Wattensburg | 1852 | |
| SPB | | S2.111 | 111 | Switzerland | Winterthur | 1850 | |
| SPB | | S2.113 | 113 | Ethiopia | Abyssinia | 1911 | |
| SPB | | S2.114 | 114 | Sardinia | Sardinia | 1911 | |
| SPB | | S2.115 | 115 | Corsica | Corsica | Not provided | |
| SPB | | S2.116 | 116 | Switzerland | Not provided | Not provided | |
| SPB | | S3.2 | 2 | Switzerland | Andeer | 2000 | |
| SPB | | S3.6 | 6 | Switzerland | Wattensburg (GR) | 1852 | |
| SPB | | S3.7 | 7 | Switzerland | Prättigau | 1850 / 1851 | |
| SPB | | S3.8 | 8 | Switzerland | Not provided | 2000 / 2001 | |
| SPB | | S3.10 | 10 | Switzerland | Glarus | 1830 | |
| SPB | | S3.15 | 15 | Switzerland | Brienzersee | 1823 | |
| SPB | | S3.17 | 17 | Switzerland | Zuoz | 1854 | |
| SPB | | S3.19 | 19 | Algeria | Algeria | 2000 | |
| SPB | | S3.27 | 27 | Ethiopia | Abyssinia | 1959 | |
| SPB | | S3.29 | 29 | Ethiopia | Abyssinia | 1959 | |
| SPB | | S3.30 | 30 | Corsica | Asco, Haute Corse | 1957 | |
| SPB | | S3.32 | 32 | Switzerland | Brig, Wallis | 1833 | |
| SPB | | S3.35 | 35 | Switzerland | Oberland, GR | 1850 | |
| SPB | | S3.36 | 36 | Switzerland | Switzerland | 1881 | |
| SPB | | S3.39 | 39 | Switzerland | Switzerland | 1891 | |
| SPB | | S3.45 | 45 | Spain | Sierra nevada | 1879 | |
| SPB | | S3.49 | 49 | Ethiopia | Abyssinia | 1990 | |
| SPB | | S3.53 | 53 | Spain | Sierra, Spain | 1899 | |
| SPB | | S3.55 | 55 | Greece | Greece | 1899 | |
| SPB | | S3.58 | 58 | Switzerland | Lausanne, Wallis | 1886 | |
| SPB | | S3.60 | 60 | Switzerland | Zermatt, Wallis | 1839 | |
| SPB | | S3.62 | 62 | Switzerland | Not provided | 1886 | |
| SPB | | S3.65 | 65 | Spain | Benasque | 1966 | |
| SPB | | S3.69 | 69 | Sardinia | Sardinia | 1915 | |
| SPB | | S3.72 | 72 | Greece | Greece | 1904 | |
| SPB | | S3.74 | 74 | Caucasus | Caucasus, North | 1900 | |
| SPB | | S3.77 | 77 | Switzerland | Tessin | 1903 | |
| SPB | | S3.87 | 87 | Spain | South Spain "gaitanes" | 1869 | |
| SPB | | S3.88 | 88 | Greece | Greece | 1904 | |
| SPB | | S3.90 | 90 | Pyrenees | Pyrenees | 1904 | |
| SPB | | S4.97 | 97 | Switzerland | Ftan, Val Tasua | 1859 | |
| SPB | | S4.99 | 99 | India | Kashmir, Ladakh | 1929 | |
| SPB | | S4.100 | 100 | India | Kashmir, Ladka | 1930 | |
| SPB | | S4.101 | 101 | Sardinia | Sardinia | 1905 | |
| SPB | | S4.105 | 105 | Greece | Smolika | 1921 | |
| SPB | | S4.106 | 106 | Sardinia | Sardegna | 1901 | |
| SPB | | S4.107 | 107 | Sardinia | Sardegna | 1907 | |
| SPB | | S4.108 | 108 | Turkestan | Naryn | 1910 | |
| SPB | | S4.109 | 109 | Central Asia | Caucasus | 1905 | |
| SPB | | S4.120 | 120 | Caucasus | Dagestan | 1900 | |
| SPB | | S4.122 | 122 | Sardinia | Not provided | Not provided | |
| SPB | | S4.125 | 125 | Sardinia | Uzulei | 1911 | |
| SPB | | S4.130 | 130 | Central Asia | Not provided | Not provided | |
| SPB | | S4.133 | 133 | Turkestan | Naryn | 1913 | |
| SPB | | S4.138 | 138 | Sardinia | Ogliastra | 1902 | |
| SPB | | S4.145 | 145 | Sardinia | Sardinia | 1901 | |
| SPB | | S4.147 | 147 | Sardinia | Sardinia | 1885 | |
| NHMT | | T1 | 1905.12.31.62 | China | Tibet | 1905 | |
| NHMT | | T2 | 1965.M.1301 | India | India | 1965 | |
| NHMT | | T3 | 1948.19.369 | India | India | 1948 | |
| NHMT | | T4 | 1948.19.368 | India | India | 1948 | |
| NHMT | | T6 | 1939.12.9.3409 | Algeria | Algeria | 1939 | |
| NHMT | | T7 | 1939.12.9.3408 | Algeria | Algeria | 1939 | |
| NHMT | | T8 | 1912.10.15.167 | Ethiopia | Abyssinia | 1912 | |
| NHMT | | T9 | 1912.10.15.165 | Ethiopia | Abyssinia | 1912 | |
| NHMT | | T10 | 1912.10.15.166 | Ethiopia | Abyssinia | 1912 | |
| NHMT | | T11 | 1912.1.28.115 | Yemen | Yemen | 1912 | |
| NHMT | | T12 | 1912.1.28.116 | Yemen | Yemen | 1912 | |
| NHMT | | T13 | 1912.1.28.117 | Yemen | Yemen | 1912 | |
| NHMT | | T14 | 1912.1.28.118 | Yemen | Yemen | 1912 | |
| NHMT | | T15 | 1912.1.28.119 | Yemen | Yemen | 1912 | |
| NHMT | | T16 | 1924.7.14.4 | Ethiopia | Abyssinia | 1924 | |
| NHMT | | T17 | 1927.9.21.1 | Ethiopia | Abyssinia | 1927 | |
| NHMT | | T18 | 1927.9.21.2 | Ethiopia | Abyssinia | 1927 | |
| NHMT | | T19 | 1934.12.16.156 | Ethiopia | Abyssinia | 1934 | |
| NHMT | | T20 | 1900.1.3.261 | Ethiopia | Abyssinia | 1900 | |
